# Affective and Cognitive Empathy in Pre-teachers With Strong or Weak Professional Identity: An ERP Study

**DOI:** 10.3389/fnhum.2019.00175

**Published:** 2019-05-31

**Authors:** Juncheng Zhu, Xin Qiang Wang, Xiaoxin He, Yuan-Yan Hu, Fuhong Li, Ming-Fan Liu, Baojuan Ye

**Affiliations:** ^1^School of Psychology, Jiangxi Key Laboratory of Psychology and Cognition Science, Center for Mental Health Education and Research, Jiangxi Normal University, Nanchang, China; ^2^Jiangxi College of Foreign Studies, Nanchang, China; ^3^Chongqing University of Arts and Sciences, Chongqing, China

**Keywords:** ERP, N110, P300, pain empathy, pre-teachers, professional identity

## Abstract

Pain empathy is influenced by a number of factors. However, few studies have examined the effects of strength of professional identity on pain empathy in pre-service teachers. This study used the event-related potential (ERP) technique, which offers a high temporal resolution, to investigate the neurocognitive mechanisms of pain empathy in pre-teachers with strong or weak professional identity. The N110 and P300 components have been shown to reflect an individual’s emotional sharing and cognitive evaluation in pain empathy, respectively. The results of the current study show that pre-teachers with strong professional identity showed a significant difference in N110 amplitudes evoked towards painful and non-painful stimuli; whereas pre-teachers with weak professional identity did not show a significant difference in the amplitudes evoked by the two stimulus types. For the P300 component, pre-teachers with weak professional identity showed a significant difference in the amplitudes evoked towards painful and non-painful stimuli; whereas pre-teachers with strong professional identity did not show a significant difference in the amplitudes evoked by the two stimulus types. Our results indicate that pre-teachers with strong professional identity show a higher level of pain empathy than those with weak professional identity.

## Introduction

Teacher empathy is a teacher’s ability to genuinely consider issues from a student’s point of view. It is the ability to see from a student’s perspective and to empathize with the student’s thoughts and feelings, thereby gaining the ability to choose suitable teaching methods and more effectively guide students in their academic and emotional growth (Peart and Campbell, 1999; Li et al., 2015). Teacher empathy is a key personal competency and an important criterion for successful vocational teaching. Studies have shown that success in vocational teaching requires the joint effects of cognitive and affective empathy (Stojiljković et al., 2012). Affective empathy enables individuals to exhibit more altruistic behaviors, whereas cognitive empathy allows individuals to rationally select the best way to help others (Smith, 2006). Teacher empathy is a key feature of teachers who have strong professional identity, allowing them to effectively establish good teacher-student relationships and a relaxed teaching environment (Stojiljković et al., 2011). It can also promote students’ academic achievement and teachers’ professional growth (Li et al., 2015; Peck et al., 2015). It is unknown whether professional identity plays a role in teacher empathy, and we thus sought to examine the effects of professional identity on teacher empathy in this study.

Pain empathy is the perception, judgment and emotional response to pain in others (Danziger et al., 2006; Meng et al., 2012) and has been shown to be one of the main manifestations of empathy in teachers (McAllister and Irvine, 2002). Studies have found that individuals may feel pain when observing pain in others, leading to greater compassion and concern (Singer et al., 2004; Gao et al., 2015). Event-related potential (ERP) studies have shown that viewing pictures of others in painful and non-painful situations leads to significant differences in the amplitudes of N110 and P300 components, with painful images evoking higher positive amplitudes (Fan and Han, 2008; Decety et al., 2010). The N110 and P300 components, which are important ERP indicators of pain empathy, have been shown to reflect an individual’s emotional sharing and cognitive evaluation in pain empathy, respectively (Fan and Han, 2008; Decety et al., 2010; Meng et al., 2013). In this study, N110 and P300 were used as key reference indicators to evaluate the differences in emotional sharing and cognitive evaluation in pre-teachers with differing levels of professional identity.

Pain empathy is influenced by a number of factors, such as attention (Gu and Han, 2007; Fan and Han, 2008), personal characteristics (Singer et al., 2006; Singer and Lamm, 2009), gender (Han et al., 2008), and attitude (Decety et al., 2009). Researchers have found that prosocial characteristics can also influence pain empathy. For example, one study in nurses found that burnout and empathy were negatively correlated in the nursing profession (Yuguero et al., 2017), suggesting that weak professional identity affects empathic abilities in nurses. Several surveys have reported that both pre-service and in-service nurses and doctors show significant positive correlations between professional identity and empathy and its components (such as perspective taking; Zhang, 2014; Mao, 2017; Visser et al., 2018). In a 10-week empathy training experiment in secondary vocational nursing students, Zhu (2017) showed that combining both traditional teaching and an experiential training model resulted in significantly improved professional identity following empathy training, particularly in the fields of professional emotion and professional expectations.

Similarly, empathy plays an important role in the teachers’ professional identity and professional development (McAllister and Irvine, 2002; Kitchen, 2005). Strong professional identity in pre-teachers enables greater empathy towards students, and hence pre-teachers with a strong professional identity are better able to adopt more suitable methods to understand and care for their students (Barr, 2011). In contrast, weak professional identity in pre-teachers may possibly lead to greater burnout, which may also significantly impact the ability of pre-teachers to empathize with their students (Kremer and Hofman, 1985; Chen, 2007). This, in turn, may hinder the healthy development of students. One narrative research study reported that empathy has a positive influence on the professional identity of teachers (Glazzard and Dale, 2013). Another survey study found that teacher professional identity is closely related to empathy and that there is an especially close relationship between professional efficacy and empathy (Goroshit and Hen, 2016). According to a core two-factor model of professional identity, professional efficacy is the core component of professional identity in teachers (Wang et al., 2011). In addition, qualitative research has found that the cultivation of strong professional identity is an important personal factor in the formation of teacher empathy (Guo, 2015). Therefore, the following hypothesis was examined in the current study: pre-teachers with stronger professional identities will show better empathic abilities, while pre-teachers with weak professional identities will show a lower level of empathy towards students.

Thus far, most previous studies on empathy have employed questionnaire surveys and behavioral methods, but few have used the ERP technique, with its high temporal resolution, to explore the neurocognitive mechanisms underlying the empathy of pre-teachers with different levels of professional identity. Investigating the neurocognitive mechanisms of pain empathy responses in pre-teachers with different levels of professional identity will help to further our understanding of the cognitive processing and neurocognitive basis of pain empathy in this group. More complete knowledge of the mechanisms of pain empathy can also provide insight into the neurocognitive mechanisms underlying the relationship between teacher professional identity and empathy. It is possible that the empathic ability of pre-teachers can be enhanced by increasing professional identity or, conversely, that developing teacher empathy can promote professional identity.

## Materials and Methods

### Participants

In China, pre-service teachers are university students who are majoring in normal education, usually at a normal university (teachers’ university) oriented to the teaching profession. These students are trained in teaching skills and participate in school-based field experiences. The Professional Identification Scale for Normal Students (PISNS; Wang et al., 2010, 2017) was administered to 395 pre-service teachers from a normal university in Jiangxi, China. Each participant was a second-semester sophomore. Pre-service teachers who scored in the top and bottom 27% were classified as belonging to the strong and weak professional identity groups, respectively. Of these, pre-service teachers were selected for inclusion in this study if they met the other inclusion criteria (e.g., voluntary participation, no previous participation in similar experiments, etc.). Women are known to occupy a larger proportion of the new generation of teachers (i.e., those who are less than 30 years of age). Especially in the lower grades, the vast majority of teachers are female (OECD, 2017). In normal universities in China, female students account for more than two-thirds of pre-service teachers (Zhu and Wang, 2017). In Chinese society and culture, it is more common for women to become teachers as this career conforms to both societal and family expectations of women. Studies have found that teachers also conform to the requirements of female students in their own professional orientation (Wang et al., 2014; Wu, 2018). Therefore, female pre-service teachers are more suitable for random samples, and the current study selected female pre-service teachers for inclusion in our sample. Of the 26 female pre-service teachers who met the inclusion criteria, two were excluded due to too many artifacts. Thus, a total of 24 female pre-service teachers were included in this study: 12 in the strong professional identity group (questionnaire total score ≥53 points, mean score: 51.33 ± 1.87) and 12 in the weak professional identity group (questionnaire total score ≤34 points, mean score: 33.33 ± 2.15). The *PISNS* total scores of the two groups were analyzed using an independent samples *t*-test, with the results showing that *t*_(22)_ = 21.88, *p* < 0.001, and *Cohen’s d* = 9.32. This implies that participant screening was effective. The participants were between the ages of 19 and 21 years, with a mean age of 19.9 years and standard deviation of 0.65 years. There was no significant difference in the mean ages of the two study groups (strong professional identity group: 19.83 ± 0.72, weak professional identity group: 20.0 ± 0.60, *t*_(22)_ = 0.62, *p* = 0.54). All participants were right-handed, had normal vision or normal corrected vision, no partial or total color blindness, no major physical or psychological diseases, and had never participated in similar experiments. The participants were informed of the purpose of the study prior to the experiment, and written informed consent was obtained. This study was approved by the ethics committee at our institution. Before the experiment, the participants did not know the reward when they finished the experiment. Participants were given a reward (15RMB and extra credit) following completion of this experiment.

### Materials

#### Professional Identification Scale for Normal Students (PISNS)

The *PISNS*, compiled by Wang et al. (2010) was used to measure the professional identity of student teachers. This scale has been used in previous studies (Wang et al., 2017; Zhu and Wang, 2017) and includes four dimensions: professional willingness and expectations, professional volition, professional values, and professional efficacy. A total of 12 items are scored on a five-point scale from 1 (strongly disagree) to 5 (strongly agree). A higher score indicates that the student teacher has a stronger professional identity. In this study, the internal consistency reliability of the scale was 0.84. Confirmatory factor analysis showed that *χ*^2^/*df* = 5.39, RMSEA = 0.08; and that model IFI, NFI, TLI, CFI and other relative fit indices all fell within an acceptable range, between 0.91 and 0.94. Thus, the overall quality of this scale is good, and it has high reliability and validity.

#### Experimental Materials in the Pain Empathy Task

The participants viewed 120 pictures, 60 of which were painful and 60 of which were non-painful. These stimuli have been used in previous ERP studies (Meng et al., 2013; Wang et al., 2014). The stimuli were all based on events in everyday life. Painful pictures showed events such as accidentally cutting one’s hand with a knife, while non-painful pictures showed events such as cutting a watermelon. The size and pixel resolution of all pictures were 9 × 6.76 cm (width × height) and 100 pixels. During the task, the pictures were presented in the center of the screen, and the size of the pictures presented was 22.5 × 16.9 cm (width × height). The distance between the pictures and the participants was 100 cm, and the viewing angle was 12.8° × 7.7°.

### Experimental Design

A 2 (professional identity: strong vs. weak) × 2 (stimulus: pain vs. no-pain) mixed factorial design was employed in this experiment, where professional identity was the between-group factor and stimulus type was the within-subjects factor. The dependent variables were the behavioral reaction time (RT) and the electroencephalography (EEG) results.

### Experimental Procedure

**Figure 1:**
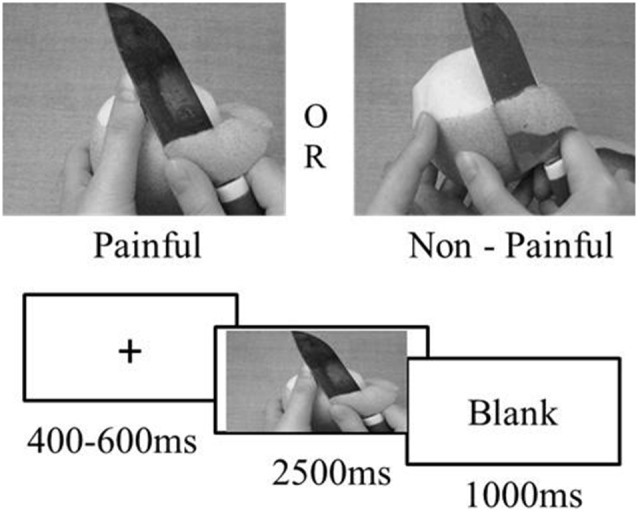
Experimental procedure.

The experimental stimuli were presented using E-Prime 2.0. The background color of the stimulus presentation was gray. The participants’ RT and correct response rate (CRR) was automatically recorded by a computer. The experiment had a total of four blocks, with 60 trials per block and a break between each block. The subjects were given the following instructions in their native language: “Please imagine that the hands and feet in the pictures are the hands and feet of your students when perceiving the following pictures.” The participants were given a practice stage prior to the start of the experiment, which allowed them to familiarize themselves with the experimental task and keypress response. First, the participants were asked to focus on the fixation cross “+” on the screen, which was followed by the stimulus pictures. If the participants perceived that pain was felt in the pictures, they were asked to press “1” on the keyboard; if not, they were asked to press “2.” Painful and non-painful pictures were presented in a random order. The fixation cross was presented for a random duration between 400 ms and 600 ms. The stimulus was then presented for a maximum duration of 2,500 ms, followed by a blank screen for 1,000 ms. The participants were required to respond as quickly and as accurately as possible. The experimental procedure is shown in Figure 1.

### Data Collection

BrainVision EEG recording and analysis software (Germany) was employed. EEG was performed using a 64-channel EEG cap, and the electrodes were distributed according to the International 10-20 system. The bilateral mastoids were used as a reference for recording. The electrodes placed on the bilateral outer canthi were used to measure horizontal electrooculography, and the electrodes placed above and below the right eye were used to measure vertical electrooculography. The impedance of all electrodes was below 5 kΩ, the bandpass filter was 0.1–30 Hz, and all EEG signals with electrode voltage greater than ±80 μv were automatically discarded (Xie et al., 2017). Fifty-two trials were discarded under each condition.

### Data Processing and Analysis

BrainVision Analyzer 2.1 was used to perform offline referencing, filtering (criteria: 0.01–30 Hz), removal of ocular interference, segmentation (−200 to 1,000 ms), baseline correction (criteria: −200 to 0 ms), artifact removal (criteria: ±80 μv), and overlaying of ERPs produced by correct responses to the target stimuli. Based on the aims of this study and previous findings on empathy, we processed and analyzed the mean amplitudes of N110 (90–160 ms) and P300 (300–460 ms). According to previous studies (Fan and Han, 2008; Han et al., 2008; Decety et al., 2010; Gao et al., 2015; Cui et al., 2016), data analysis was performed using the F3, F4, FZ, FC3, FC4 and FCZ electrodes for the N110 component and the P3, P4, PZ, PO3, PO4 and POZ electrodes for the P300 component. A 2 (professional identity: strong vs. weak) × 2 (stimulus type: pain vs. no-pain) × (electrode position) three-way repeated measures analysis of variance (ANOVA) was performed, and a Greenhouse-Geisser correction was performed on the resulting *p* values. The raw data supporting the conclusions of this manuscript will be made available by the authors, without undue reservation, to any qualified researcher.

## Results

### Behavioral Results

The mean RT and CRR of participants with strong and weak professional identities are shown in Table 1. Two-way repeated measures ANOVA was performed on the RT and CRR. For RT, the main effect of stimulus types was not significant (*F*_(1,22)_ = 1.16, *p* > 0.05); the main effect of professional identity was not significant (*F*_(1,22)_ = 1.70, *p* > 0.05); and the interaction effect of stimulus type and professional identity was not significant (*F*_(1,22)_ = 1.59, *p* > 0.05). Likewise, for CRR, the main effect of stimulus types was not significant (*F*_(1,22)_ = 2.58, *p* > 0.05); the main effect of professional identity was not significant (*F*_(1,22)_ = 0.51, *p* > 0.05); and the interaction effect of stimulus type and professional identity was not significant (*F*_(1,22)_ = 0.19, *p* > 0.05).

**Table 1:** Mean reaction time (RT) and correct response rate (CRR) of the pain empathy task (M ± SD).

	Strong professional identity (*n* = 12)		Weak professional identity (*n* = 12)	
	Pain	No-pain	Pain	No-pain
RT (ms)	970.22 ± 138.61	873.95 ± 139.17	920.54 ± 127.82	877.82 ± 155.15
CRR (%)	0.91 ± 0.08	0.93 ± 0.04	0.92 ± 0.09	0.96 ± 0.07

### ERP Results

**Figure 2:**
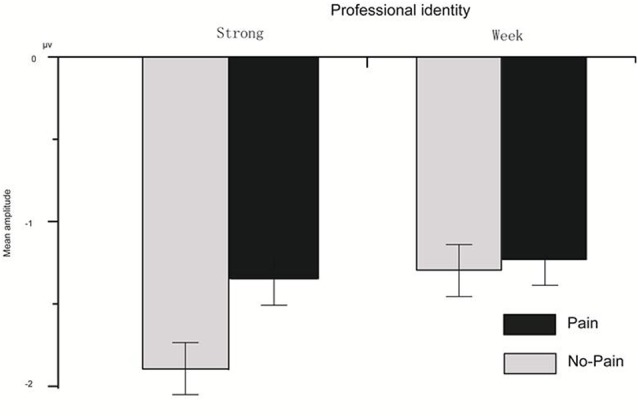
Interaction effect of professional identity and pain empathy for the N110 component.

For the N110 component, the main effect of N110 component amplitude was significant (*F*_(1,22)_ = 3.64, *p* = 0.02, $\eta_{\text{p}}^{2}$ = 0.50); the main effect of stimulus type was significant (*F*_(1,22)_ = 8.63, *p* = 0.008, $\eta_{\text{p}}^{2}$ = 0.28); the main effect of professional identity was not significant (*F*_(1,22)_ = 0.26, *p* = 0.61); the interaction effect of the amplitude and stimulus type was not significant (*F*_(1,22)_ = 0.84, *p* = 0.54); the interaction effect of the amplitude and stimulus type and professional identity was not significant (*F*_(1,22)_ = 0.76, *p* = 0.59); and the interaction effect of stimulus type and professional identity was marginally significant (*F*_(1,22)_ = 3.94, *p* = 0.06, $\eta_{\text{p}}^{2}$ = 0.15). *P* < 0.05 is not an absolute threshold of rejecting or accepting the hypothesis, and to ignore marginally significant findings may overlook the important results tightly associated with research questions. We have a clearly defined research hypothesis about the association between professional identity and stimulus type, and it is thus worthy to fully investigate the simple main effects even if the ANOVA interaction is only marginally significant. Simple effects testing of the interaction effect between stimulus type and professional identity showed that in the strong professional identity group there was a significant difference between painful and non-painful stimuli (*p* = 0.001; Figure 2; for FZ in Figure 4). Further, painful stimuli evoked a less negative N110 amplitude than did non-painful stimuli. In the weak professional identity group, there was no significant difference between painful and non-painful stimuli (*p* = 0.52).

**Figure 3:**
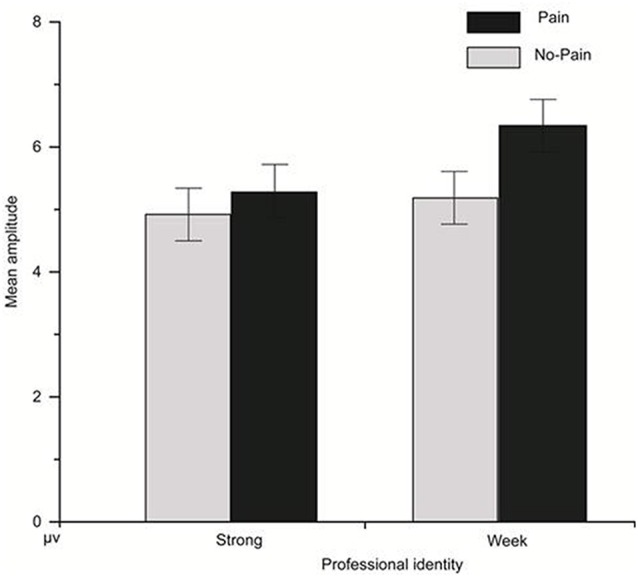
Interaction effect of professional identity and pain empathy for the P300 component.

**Figure 4:**
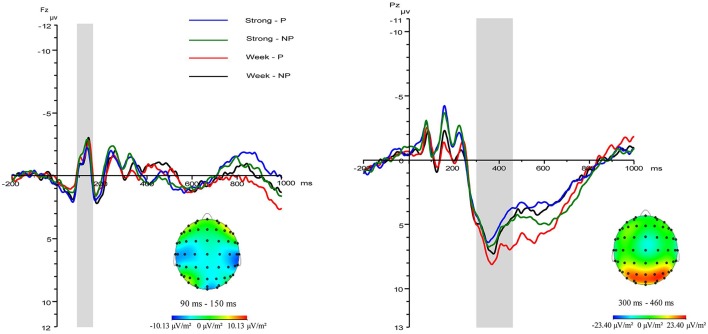
Grand mean waveforms of event-related potential (ERP) evoked by painful and non-painful stimuli in pre-teachers with different levels of professional identity. Strong-P/NP represent painful and non-painful stimulus, respectively, in participants with strong professional identity; Weak-P/NP represent painful and non-painful stimulus, respectively, in participants with weak professional identity.

For the P300 component, the main effect of P300 component amplitude was significant (*F*_(1,22)_ = 6.60, *p* = 0.001, $\eta_{\text{p}}^{2}$ = 0.65); the main effect of stimulus type was significant (*F*_(1,22)_ = 10.07, *p* = 0.004, $\eta_{\text{p}}^{2}$ = 0.31); the interaction effect of the amplitude and stimulus type was significant (*F*_(1,22)_ = 9.25, *p* = 0.001, $\eta_{\text{p}}^{2}$ = 0.72); the interaction effect of the amplitude and stimulus type and professional identity was not significant (*F*_(1,22)_ = 0.63, *p* = 0.68); the main effect of professional identity was not significant (*F*_(1,22)_ = 0.73, *p* = 0.40); and the interaction effect of stimulus type and professional identity was marginally significant (*F*_(1,22)_ = 3.66, *p* = 0.07, $\eta_{\text{p}}^{2}$ = 0.14). *P*<0.05 is not an absolute threshold of rejecting or accepting the hypothesis, and to ignore marginally significant findings may overlook the important results tightly associated with research questions. We have a clearly defined research hypothesis about the association between professional identity and stimulus type, and it is thus worthy to fully investigate the simple main effects even if the ANOVA interaction is only marginally significant. Simple effects testing of the interaction effect between stimulus type and professional identity showed that, in the strong professional identity group, there was no significant difference between painful and non-painful stimuli (*p* = 0.36). In the weak professional identity group, there was a significant difference between painful and non-painful stimuli (*p* = 0.002), with painful stimuli evoking a more positive P300 amplitude than non-painful stimuli shown in Figure 3; for PZ in Figure 4.

For the frontal section of the brain, we chose four electrodes (F3, F4, FC3, and FC4) and analyzed 2 hemispheres (left, right) × 2 groups (professional identity: strong vs. weak) × 2 experimental conditions (stimulus type: pain vs. no-pain). According to the results of the repeated measures ANOVA, the main effect of hemisphere was not significant (*F*_(1,22)_ = 0.43, *p* = 0.52); the interaction effect of hemisphere and professional identity was not significant (*F*_(1,22)_ = 0.003, *p* = 0.96); and the interaction effect of hemisphere and stimulus type and professional identity was not significant (*F*_(1,22)_ = 1.86, *p* = 0.19).

For the parietal section of the brain, we chose four electrodes (P3, P4, PO3, PO4) and analyzed 2 hemispheres (left, right) × 2 groups (professional identity: strong vs. weak) × 2 experimental conditions (stimulus type: pain vs. no-pain). According to the results of the repeated measures ANOVA, the main effect of hemisphere was not significant (*F*_(1,22)_ = 0.27, *p* = 0.61); the interaction effect of hemisphere and professional identity was not significant (*F*_(1,22)_ = 0.08, *p* = 0.78); and the interaction effect of hemisphere and stimulus type and professional identity was not significant (*F*_(1,22)_ = 0.50, *p* = 0.49).

## Discussion

To the best of our knowledge, this is the first ERP study to examine the effects of strength of professional identity on pain empathy in pre-service teachers. This study examined the cognitive processing features of pain empathy in pre-service teachers with strong and weak professional identities using the ERP technique, which has a high temporal resolution. Our results revealed that, although behavioral indicators showed no significant differences in the pain empathy between pre-teachers with strong professional identity and those with weak professional identity, painful pictures evoked less negative amplitudes for the N110 and P300 components in both groups. This finding is consistent with past studies (Fan and Han, 2008; Meng et al., 2012, 2013), indicating that the experimental manipulation of this study was valid. The results of the current study also demonstrate that pre-teachers with strong professional identity showed a significant difference in the N110 component when shown painful vs. non-painful stimuli, whereas pre-teachers with weak professional identity did not show a significant difference. In contrast, pre-teachers with weak professional identity showed a significant difference in the P300 component when shown painful vs. non-painful stimuli, whereas pre-teachers with strong professional identity did not show a significant difference.

The N110 component reflects an individual’s early perceptual processing and is a key indicator for the mechanisms of emotional sharing in pain empathy (Fan and Han, 2008; Han et al., 2008; Decety et al., 2010). Our results indicate that there is a significant difference in the N110 component evoked by painful vs. non-painful stimuli in pre-teachers with strong professional identity, but not in those with weak professional identity. According to the theory of emotional sharing, the basis of empathy is the emotional sharing between individuals (Jeannerod, 1999; Decety and Sommerville, 2003). Individuals with stronger empathic abilities will thus also show greater emotional sharing (Decety and Lamm, 2006). Two key features of pre-teachers with strong professional identity are their strong identification with the teaching profession and their high enthusiasm towards their students. Pre-teachers with strong professional identity were able to engage in different levels of emotional sharing when faced with painful and non-painful stimuli. This implies that they are more easily affected by painful stimuli experienced by students, which evokes similar perceptions of pain. Conversely, pre-teachers with weak professional identity would be expected to show low identification with the teaching profession and low enthusiasm towards their students. As expected, the results of the current study demonstrate that pre-teachers with weak professional identity did not show significant differences in emotional sharing between painful and non-painful stimuli. Taken together, these results imply that pre-teachers with different levels of professional identity have different thresholds of empathy for student pain.

The P300 component reflects an individual’s cognitive evaluation and regulatory processing of pain empathy. It occurs after the N110 component and involves the conscious cognitive evaluation of stimuli (Fan and Han, 2008; Han et al., 2008; Song et al., 2016). Decety et al. (2010) compared the differences in pain empathy between doctors and ordinary individuals and found no significant difference in the P300 components induced by doctors’ observations of pain vs. non-pain pictures. This reflects the importance of doctors being trained to reduce and ignore the disturbance and inner impact caused by the perception of pain in order to maintain professional behavior. That is to say, doctors should adjust and suppress negative emotions induced by pain stimuli in order to focus their cognitive resources on helping others. Our results demonstrate that pre-teachers with weak professional identity show significant differences in the P300 amplitudes evoked by painful and non-painful stimuli, whereas this difference was not significant in pre-teachers with strong professional identity. Empathy can be subdivided into affective and cognitive empathy (Stojiljković et al., 2012). Although teachers are as helpful as doctors, teachers should not ignore the suffering of students, but should always care about the suffering of students, and show empathy. The N110 component reflects early perception processing and is an important indicator of emotional sharing in pain empathy. This study found that there were significant differences in the N110 component between pre-service teachers with strong professional identity under pain and non-pain stimuli. This result suggests that pre-teachers with strong professional identity show greater abilities in emotional sharing and that these same pre-teachers are better able both to recognize the reasons for empathizing with their students and to attenuate the arousal level elicited by painful stimuli, leading to a lowered amplitude of the P300 component. Pre-teachers with strong professional identity show emotional sharing in pain empathy at an earlier point in perceptual processing, which may facilitate the regulation and alleviation of emotional exhaustion. As for pre-teachers with weak professional identity, the significant difference in the P300 component when shown painful vs. non-painful stimuli is due to their lower emotional sharing abilities towards their students and the activation of the internal aversive motivational system by the negative stimuli (Bartholow et al., 2006). This in turn led to higher arousal levels towards painful stimuli. In conclusion, pre-teachers with stronger professional identities showed better empathic abilities, as measured by ERP, while pre-teachers with weak professional identities showed a lower level of pain empathy. Studies have found that empathy in teachers can be significantly improved through training (Warner, 1984). Therefore, in the future, researchers may consider improving professional identity and alleviating job burnout by training teachers in empathy. The results of this study support the results of previous qualitative and investigative studies that have reported a close correlation between teacher professional identity and empathy (Glazzard and Dale, 2013; Li et al., 2015; Goroshit and Hen, 2016). However, because relatively few participants volunteered and met the inclusion criteria for the present study, our results need to be confirmed in future studies using larger samples. In addition, this research also has some limitation, such as simple effects testing after the interaction effect of stimulus type and professional identity was marginally significant. Some studies have suggested that social relationships and interpersonal distance affect pain empathy (Song et al., 2016; Cross et al., 2019). However, participants are a second-semester sophomore in present study, who are mainly in the learning stage of theoretical knowledge and skills of education and teaching, and have a low degree of real involvement in education and teaching and contact with students, which may be the potential causes of the marginal significance. In the future, senior or in-service teachers with deeper socialization of teacher-student relationship could be chosen to further explore the interaction effect of stimulus type and professional identity.

## Ethics Statement

This study was carried out in accordance with the recommendations of the ethics committee of School of Psychology, Jiangxi Normal University. The protocol was approved by the ethics committee of School of Psychology, Jiangxi Normal University.

## Author Contributions

XW was responsible for the design of the study, data collection, interpretation of data for the work, article writing and revising. JZ contributed to the design of the study, data collection, data analysis, article writing and revising. XH contributed towards data collection. YH, FL, ML, and BY all contributed towards the revision of the article.

## Conflict of Interest Statement

The authors declare that the research was conducted in the absence of any commercial or financial relationships that could be construed as a potential conflict of interest.
